# Apoptotic bodies from endplate chondrocytes enhance the oxidative stress‐induced mineralization by regulating PPi metabolism

**DOI:** 10.1111/jcmm.14268

**Published:** 2019-03-20

**Authors:** Feng‐Lai Yuan, Rui‐Sheng Xu, Jun‐Xing Ye, Ming‐Dong Zhao, Li‐Jun Ren, Xia Li

**Affiliations:** ^1^ Department of Orthopaedics and Central Laboratory The Third Hospital Affiliated to Nantong University Wuxi China; ^2^ Department of Orthopaedics and Central Laboratory The Hospital Affiliated to Jiangnan University Wuxi China; ^3^ Department of Orthopaedics, Jinshan Hospital Fudan University Shanghai China; ^4^ Department of Medicine Anhui College of Traditional Chinese Medicine Wuhu China

**Keywords:** apoptotic bodies, endplate chondrocytes, intervertebral disc degeneration, mineralization

## Abstract

This study aimed to investigate the role of apoptotic bodies (Abs) from the oxidative stressed endplate chondrocytes in regulating mineralization and potential mechanisms. Endplate chondrocytes were isolated from rats and treated with H2O2 to induce oxidative stress. The calcium deposition for matrix mineralization in the cells was examined by histological staining. The expression levels of calcification‐related genes in individual groups of cells were determined by quantitative real time‐PCR (qRT‐PCR). Subsequently, extracellular vesicles (EVs) were purified and characterized. The effect of treatment with H2O2 and/or Abs on the mineralization, extracellular PPi metabolism and related gene expression were determined. Oxidative stress significantly increased the mineralization and promoted the generation of main Abs from endplate chondrocytes. Abs were effectively endocytosed by endplate chondrocytes and co‐localized with collagen (COL)‐II in the cytoplasm, which enhanced the mineralization, alkaline phosphatase (ALP), osteocalcin (OCN), Runt‐related transcription factor 2 (RUNX2) and COL‐I expression in endplate chondrocytes. Furthermore, treatment either H2O2 or Abs significantly decreased PPi, but increased Pi production and treatment with both further enhancing the changes in endplate chondrocytes. Similarly, treatment either H2O2 or Abs significantly decreased the ectonucleotide pyrophosphatase/phosphodiesterase 1 (ENPP1), and ankylosis protein (ANK) expression and ENPP1 promoter activity, but increased the tissue‐nonspecific alkaline phosphatase (TNAP) expression and TNAP promoter activity in endplate chondrocytes. Oxidative stress promoted the generation of Abs, which might enhance the oxidative stress‐mediated mineralization in endplate chondrocytes by regulating the PPi metabolism.

## 1 INTRODUCTION

Low back pain is a major cause of work‐related disabilities worldwide and causes significanthealthcare‐related costs.^1^ A leading cause of low back pain is degeneration of the intervertebral disc (IVD).^2^ Although many factors are associated with the occurrence of IVD degeneration nutritional dysregulation is crucial for the IVD degeneration as the IVD is the largest avascular organ in the body.^3^ Foundational solutes diffusing through the cartilage endplate (CEP) is the primary route to supply nutrients to the IVD.^4^ The CEP is a thin layer of hyaline cartilage, which lies between the vertebral body and the intervertebral disc.^5^ Previous studies have shown that CEP calcification promotes the degradation of IVD because it prevents the diffusion of nutrients into the disc.^6^ Hence, understanding the mechanisms underlying CEP calcification will be of great significance in developing new therapies for prevention and intervention of IVD degeneration and low back pain.

Increased oxidative stress can cause inflammation and promote the IVD degeneration.^7^ Oxidative stress can produce excessive reactive oxygen species (ROS) and cause inflammation, which can promote catabolism and premature senescence, affect the matrix homoeostasis and induce apoptosis of IVD cells, leading to IVD degeneration.^8^ Actually, high levels of oxidative stress occur during the process of CEP degeneration, participating in the degeneration of CEP.^9^ A recent study has shown that increased oxidative stress induces apoptosis and promotes calcification in CEP cells.^10^ However, how apoptosis participates in oxidative stress‐induced calcification of CEP cells has not been clarified.

Extracellular vesicles, including exosomes (Exo), microvesicles (MVs) and apoptotic bodies (Abs) can regulate the process of calcification in the arteries and heart valves.^11^ These membrane‐bound vesicles contain the necessary calcium‐binding proteins and phosphatases for nucleation of hydroxyapatite. Actually, extracellular vesicle‐like structures have also been found in mineralizing cartilage.^12^, ^13^ These extracellular vesicles participate in cellular communication between cells in joint tissues and regulate the turnover of the extracellular matrix.^14^ It is well known that autophagy and apoptosis can increase the generation of extracellular vesicles in cells. A recent study indicates that Abs from chondrocytes may contribute to the pathogenic process of cartilage calcification in aging patients with osteoarthritis.^15^ However, it is unclear whether oxidative stress can increase the production of Abs and whether Abs can regulate the expression of calcification‐related genes, such as ALP, osteocalcin (OCN), Runt‐related transcription factor 2 (RUNX2) and collagen II (COL‐II) remain unclear. In addition, it is well known that the levels of extracellular PPi and Pi are critical for mineralization in cartilage.^15^ However, there is no information on whether Abs can regulate the levels of PPi and Pi and the expression of PPi‐related genes, such as ectonucleotide pyrophosphatase/phosphodiesterase 1 (ENPP1) and ankylosis protein (ANK) and tissue‐nonspecific alkaline phosphatase (TNAP) in primarily cultured endplate chondrocytes.

In the present study, we isolated endplate chondrocytes of the IVD of rats and explored whether increased oxidative stress could increase the production of extracellular vesicles. Subsequently, we examined the effect of Abs on mineralization, PPi metabolism and related gene expression in primarily cultured endplate chondrocytes ex vivo. Our findings indicated that increased oxidative stress promoted the generation of Abs, which enhanced the oxidative stress‐induced mineralization by modulating the PPi metabolism in endplate chondrocytes.

## 2 MATERIALS AND METHODS

### 2.1 Ethics statement

The experimental protocol was approved by the Institutional Animal Committee of Nantong University (Nantong, China, Permit Number: ntdx2017‐0082). All animals were cared, in accordance with the ‘Guidelines for the Care and Use of Laboratory Animals' as stated in the Helsinki Declaration. At the end of the experiment, the animals were killed using pentobarbital sodium.

### 2.2 Culture and treatment of endplate chondrocytes

Endplate chondrocytes were isolated from the endplate cartilage of 4‐week‐old Sprague‐Dawley (SD) rats, as described previously.^13^ Endplate chondrocytes were cultured overnight in Dulbecco's Modified Eagle's Medium (DMEM; Gibco, Grand Island, NY) supplemented with 10% foetal bovine serum (FBS; Gibco), and exposed to fresh medium every 3 days. The cells were treated with, or without, different concentrations (0‐2.0 mmol/L) H_2_O_2_ for various time periods (0‐21 days) in the FBS‐free medium.

### 2.3 Alizarin red s and von kossa staining

Formation of mineralized nodules by endplate chondrocytes in vitro was analysed by Alizarin Red S and von Kossa staining. Briefly, endplate chondrocytes were rinsed with PBS and fixed in ice‐cold 90% ethanol solution at room temperature for 15 minutes. After being washed, the cells were stained with Alizarin Red solution (Amresco, Solon, OH) for 15 minutes in the dark at room temperature. The results were expressed as the percentage of positive staining area in per field of view. For the quantitative measurement of alizarin‐red staining, the dye was dissolved in 10% cetylpyridinium chloride (Nanjing Elegant Nutraceuticals, Nanjing, China) in 10 mmol/L sodium phosphate (pH 7.0; Amresco), and the OD value at 620 nm was measured. The net OD value was the OD value of each test group—control well with no cells.

For von Kossa staining, the fixed cells in each well were stained with 5% silver nitrate solution (Sigma, USA) and exposed to UV radiation for 1.5 hour. After being washed, individual wells were added with 250 μL of sodium thiosulfate and incubated for 5 minutes to remove nonspecific staining. The percentages of von Kossa positive staining were quantified in each experimental group.

### 2.4 Alkaline phosphatase staining and alp activity assay

Mineralized nodules were detected by alkaline phosphatase (ALP) staining, according to the manufacturer's instructions (Beyotime Institute of Biotechnology, Haimen, China). The ALP activity of individual groups of cells was determined by enzymatic assay using the Alkaline Phosphatase Assay Kit (Beyotime Institute of Biotechnology) according to the manufacturer's protocol. Briefly, the individual groups of cells were stained with black cobaltous sulphide staining for ALP and counterstained with nuclear fast red, followed by imaging under a light microscope (magnification, x100; Nikon Eclipse TC 100; Nikon Corporation, Tokyo, Japan).

### 2.5 Abs Purification by centrifugation

Abs from the supernatants of culturedendplate chondrocytes were isolated by a sequential centrifugation approach.^16^ Following treatment with H_2_O_2_ [1 mmol/L]) for 12 hours to induce endplate chondrocyte apoptosis, the supernatants were subjected to multiple steps of centrifugations, first at 300 *g* for 10 minutes and 3000 *g* for 20 minutes to pellet Abs. Subsequently, the pellets were washed, resuspended and passed through a 0.8‐mm pore filter, followed by centrifuging at 16 000 *g* for 40 minutes. Finally, the resulting supernatants were passed through a 0.2‐mm pore filter, and centrifuged at 100 000 *g* for 1 hour to pellet exosomes. The concentrations of proteins in individual Abs were determined by Bradford protein assay.

### 2.6 Electron microscopy

After being washed, the H2O2‐treated endplate chondrocytes were fixed immediately in 2.5% glutaraldehyde, post‐fixed in 2% osmium tetroxide in 0.15 mol/L cacodylate buffer for 1 hour, washed in distilled water, dehydrated in a graded series of acetone series (30%‐100%) and embedded in Spurr resin, followed by ultrathin sectioning at 70 nm. The sections were stained with uranyl acetate and lead citrate and analysed by transmission electron microscopy (JEM‐1230; Jeol).

For scanning electron microscope, the samples were further fixed in 1% osmium tetroxide aqueous solution for 10 minutes and then dehydrated in a graded ethanol series (60%‐100%). The slides were covered in hexamethyldisilazane for 30 seconds, left to dry in a dessicator, sputter‐coated in gold and viewed in a scanning electron microscope (JSM‐840; Jeol).

### 2.7 Inorganic pyrophosphate and inorganic phosphate assay

The concentrations of Inorganic pyrophosphate (PPi) and inorganic phosphate (Pi) in the supernatants of cultured cells were measured by enzyme‐linked immunosorbent assay (ELISA) using the Pyrophosphate Assay Kit and Phosphate Assay Kit (Molecular Probes, USA), respectively, according to the manufacturer's instructions. The reaction mixtures were incubated in triplicate for 10 minutes at room temperature, and measured for absorbance at 360 nm.

### 2.8 Quantitative real time‐PCR analysis

The related levels of ALP, RUNX2, OCN, COL‐I, ENPP1, TNAP and ANK mRNA transcripts to the control GAPDH in individual samples were determined by quantitative real time‐PCR (qRT‐PCR) using the SYBR Green PCR Master Mix (Applied Biosystems, Foster City, CA), and specific primers, according to the manufacturer's protocols. The primer sequences are shown in Table 1.

**Table 1:** Primers for quantitative RT‐PCR

Target gene	Forward primer (5′‐3′)	Reverse primer (5′‐3′)
ALP	AACCTGACTGACCCTTCCCTCT	TCAATCCTGCCTCCTTCCACTA
Runx2	AGTAAGAAGAGCCAGGCAGGTG	GTGTAAGTGAAGGTGGCTGGATAG
OCN	GCATTCTGCCTCTCTGACCTGG	GCTCCAAGTCCATTGTTGAGGTAG
Col‐1	CTGCTGGCAAGAATGGCGA	GAAGCCACGATGACCCTTTATG
NPP1	TCAGTACCATTTGAAGAAAGGATT	GTCAGAGCCATGAAATCCACTTCC
TNAP	CTGACTGACCCTTCGCTCTC	TCATGATGTCCGTGGTCAAT
ANK	CTTCTAGCAGGGTTTGTGGG	TCGTCTCTTTCCTCCTCCTC
GAPDH	TGCTGGTGCTGAGTATGTGGT	AGTCTTCTGGGTGGCAGTGAT

### 2.9 Western blot analysis

The different groups of cells were harvested and lyzed in lysis buffer containing protease inhibitor mixtures (Roche Applied Science, Indianapolis, USA). Similarly, the nuclear and cytosolic proteins were extracted using a Nuclear/Cytosol Fractionation Kit (BioVision, Mountain View, CA) according to manufacturer's instructions. The protein concentrations were determined using a BCA‐200 protein assay Kit (Pierce, Rockford, IL). Individual cell lysates (30 μg/lane) were separated by sodium dodecyl sulphate‐polyacrylamide gel electrophoresis (SDS‐PAGE) on 12% gels and transferred onto polyvinyl difluoride (PVDF) membrane (Pierce). The membranes were blocked with 5% skimmed dry milk in TBST (10 mmol/L Tris, 150 mmol/L NaCl and 0.05% Tween 20; pH 8.3) and incubated with primary antibodies against histone H3, ARF6 or CD9 as well as GAPDH at 4°C overnight. After being washed, the bound antibodies were detected with HRP‐conjugated secondary antibodies and visualized using the enhanced chemiluminescence reagents. The relative levels of target proteins to the control were determined by densitometric analysis using a software (Gel Logic 2200; Rochester, NY).

### 2.10 Immunofluorescence staining

The intracellular distribution of Abs in endplate chondrocytes was analysed by immunofluorescence. Briefly, endplate chondrocytes were incubated with Alexa Fluor 555‐Annexin V and treated with 1 μg/mL of Abs for 30 or 60 minutes, followed by staining with FITC‐anti‐Col II (Molecular Probes). After being washed, the cells were examined under a laser scanning confocal microscope.

### 2.11 Luciferase reporter assay

ENPP1 and TNAP activities were measured using a luciferase reporter assay.^17^ Endplate chondrocytes were cotransfected with pGL3‐ENPP1‐prom or pGL3‐TNAP‐prom and Renilla luciferase reporter vector using Lipofectamine 2000 (Invitrogen) for 24 hours. The cells were treated with, or without, H_2_O_2_ or Abs for 12 hours. The cells were collected and lyzed in Lysis Buffer (Promega). The ENPP1 and TNAP activities were determined by dual luciferase assays using the dual luciferase reporter kit (Promega). Firefly luciferase activity was normalized to Renilla luciferase activity.

### 2.12 Statistical analysis

Data are expressed as mean ± standard error of mean (SEM). The difference between two groups was analysed by Student's *t* test and the difference among groups was analysed by one‐way analysis of variance (ANOVA) and post hoc least significant difference (LSD) using the SPSS software for Window version 17. A *P* < 0.05 was considered statistically significant.

## 3 RESULTS

### 3.1 Oxidative stress induces mineralization in primary endplate chondrocytes from rat IVDs

**Figure 1:**
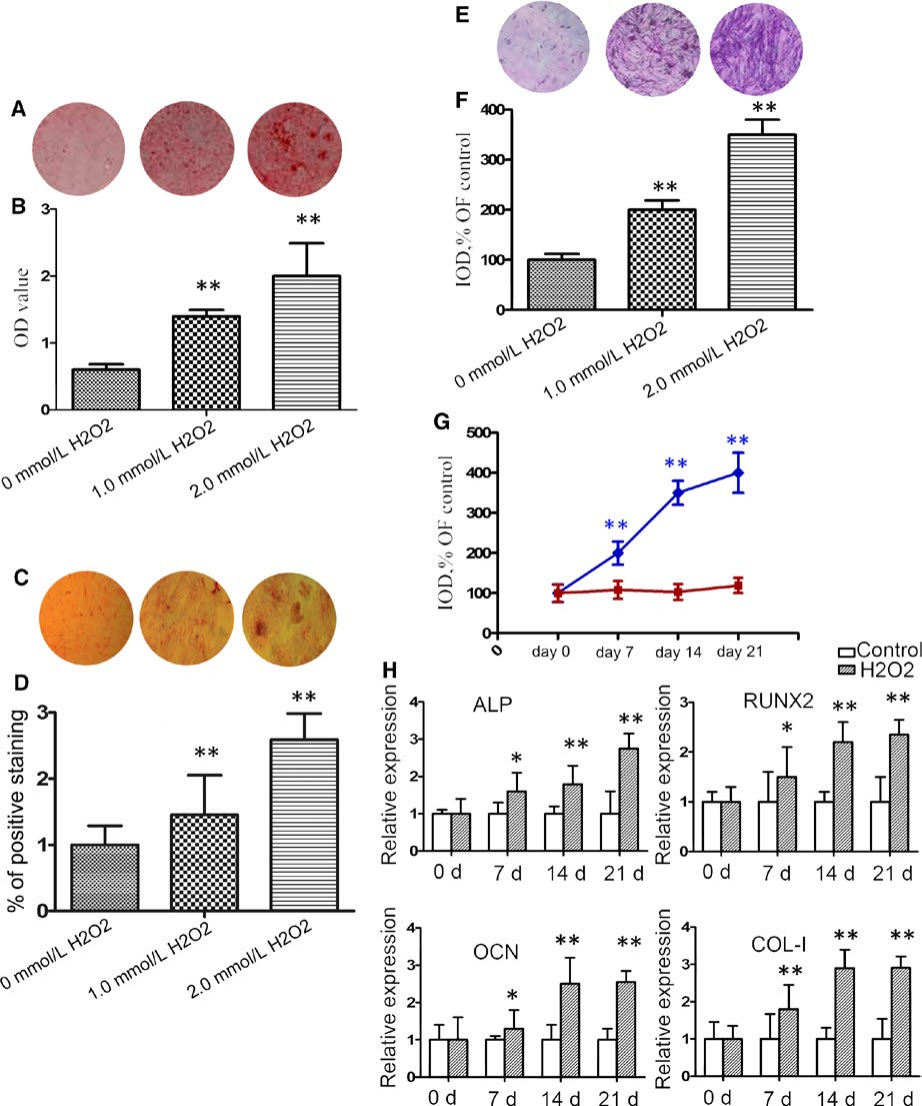
Oxidative stress induces mineralization in primary endplate chondrocytes from rat intervertebral discs (IVDs). Endplate chondrocytes were isolated from rats and treated with, or without, H_2_O_2_ at the indicated doses for 7 days. (A) Alizarin Red staining for calcium deposition in endplate chondrocytes. (B) Semi‐quantitative analysis of the mineralized nodule in endplate chondrocytes. (C) von Kossa staining. (D) The percentage of von Kossa‐positive cells. (E) ALP staining in endplate chondrocytes. (F) Semi‐quantitative analysis of alkaline phosphatase (ALP) activities in endplate chondrocytes. (G) Longitudinal analysis of ALP activities in endplate chondrocytes following treatment with 2.0 mM H_2_O_2_. (H) Quantitative real time polymerase chain reaction (RT‐PCR) analysis of the relative levels of ALP, RUNX2, OCN and COL‐I mRNA transcripts in endplate chondrocytes. Data are representative images (magnification x 100) or expressed as the mean ± SEM of each group of cells from three separate experiments. *P < 0.05, **P < 0.01 vs the control

To explore the effects of oxidative stress on mineralization of endplate chondrocytes from IVDs, endplate chondrocytes were isolated from SD rats and treated with different concentrations of H_2_O_2_, followed by Alizarin Red and von Kossa staining. As shown in Figure 1A‐D, induction of oxidative stress increased calcium and phosphate deposition for matrix mineralization in a trend of dose‐dependence in endplate chondrocytes. Similarly, treatment with H_2_O_2_ increased the ALP activities in a trend of dose‐dependence in endplate chondrocytes (Figure 1E,F). In addition, treatment with H_2_O_2_ (2.0 mmol/L) for varying periods increased the ALP activities in endplate chondrocytes in a time‐dependent manner (Figure 1G). Further qRT‐PCR assays indicated that treatment with H_2_O_2_ (2.0 mmol/L) significantly increased the relative levels of ALP, RUNX2, OCN and COL‐I mRNA transcripts to the control GAPDH in endplate chondrocytes (Figure 1H). Such data indicate that oxidative stress induces mineralization in primarily cultured endplate chondrocytes from IVDs.

### 3.2 Oxidative stress increases the generation of apoptotic bodies (Abs) by endplate chondrocytes from rat IVDs

**Figure 2:**
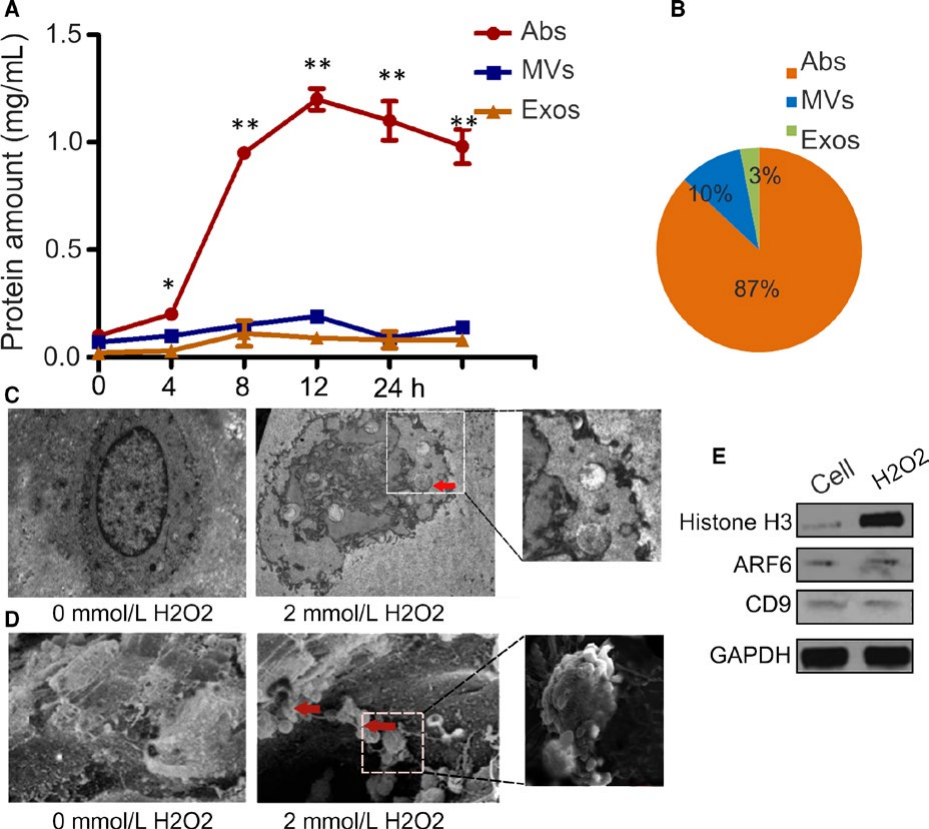
Oxidative stress promotes the generation of main Abs in endplate chondrocytes. Endplate chondrocytes were treated with, or without, H_2_O_2_ for 4, 8, 12 and 24 hours and the extracellular vesicles (EVs) in the supernatants of cultured cells were purified. (A) The levels of different types of EVs in the supernatants of cultured cells were examined longitudinally for protein contents. (B) The percentages of each type of EVs in the supernatants of cultured cells following treatment with 2.0 mmol/L H_2_O_2_. (C) Transmission electron microscopy of H_2_O_2_‐treated endplate chondrocytes. Treated cell demonstrated the presence of Abs. (D) Scanning electron microscopy of H_2_O_2_‐treated endplate chondrocytes. (E) The relative levels of Histone H3, ARF6 and CD9 to the control GAPDH expression were determined by Western blot. Data are representative images or expressed as the mean ± SEM of each group of cells from three separate experiments. *P < 0.05, **P < 0.01 vs the control

Increased oxidative stress can induce chondrocyte apoptosis.^18^, ^19^ To determine the impact of oxidative stress on the production of Abs, endplate chondrocytes were treated with, or without, H_2_O_2_ for varying time periods and the secreted EVs in the cultured cells were purified. As shown in Figure 2A, treatment with H_2_O_2_ significantly increased the levels of Abs, but only minimally increased the levels of MVs and Exos in the supernatants of cultured endplate chondrocytes. The percentages of Abs were several‐fold higher than that of others at 12 hours post‐H_2_O_2_ treatment (Figure 2B). Electron microscopy analysis revealed Abs with a size range of 1000‐3000 nm from the H_2_O_2_‐treated endplate chondrocytes (Figure 2C,D). Further Western blot analysis indicated higher levels of histone H3 expression, but little ARF6 and CD9 expression (the representative markers of MVs and Exo, respectively, Figure 2E) in the purified EVs, consistent with the character of Abs.^20^, ^21^ Hence, oxidative stress increased the production of Abs in the primary endplate chondrocytes from rat IVDs, which may be associated with mineral deposition in endplate chondrocytes.

### 3.3 Abs from the oxidative stressed endplate chondrocytes promote mineralization

**Figure 3:**
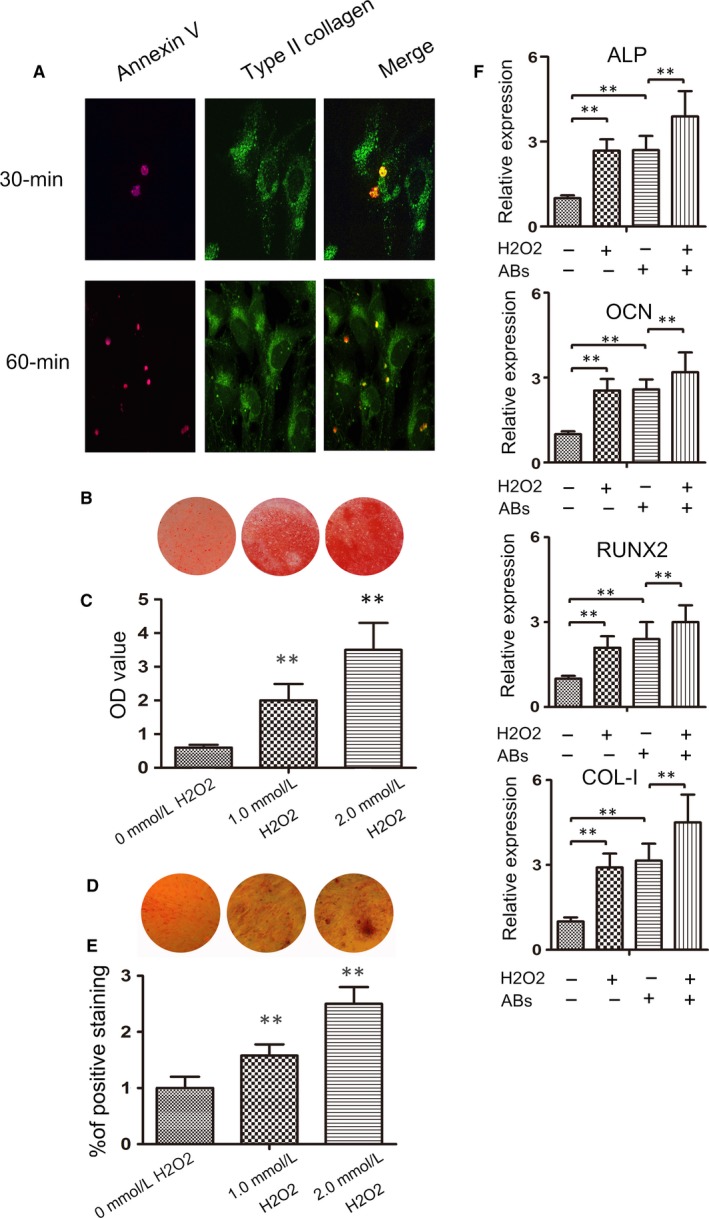
Apoptotic bodies (Abs)_ are effectively endocytosed and co‐localized with COL‐II to promote mineralization in endplate chondrocytes. Endplate chondrocytes were treated with, or without, Abs at the indicated doses for indicated time periods or treated with, or without, H_2_O_2_ and/or Abs for 7 days. (A) Subcellular distribution of Abs in endplate chondrocytes was analysed using confocal microscopy after staining with anti‐collagen II. (B) Alizarin Red S staining for calcium deposition in endplate chondrocytes. (C) Semi‐quantitative analysis of the mineralized nodules in endplate chondrocytes. (D) von Kossa staining of phosphate contents in endplate chondrocytes. (E) The percentage of von Kossa‐positive cells. (F) Quantitative RT‐PCR analysis of the relative levels of ALP, RUNX2, OCN and COL‐I to the control GAPDH mRNA transcripts in endplate chondrocytes. Data are representative images (magnification x 100) or expressed as the mean ± SEM of each group of cells from three separate experiments. **P < 0.01 vs the control

To examine the role of Abs in mineralization, endplate chondrocytes were treated with Alexa Fluor 555‐annexin V and treated with Abs for 30 or 60 minutes. The cells were stained with FITC‐anti‐Col II. The distribution of endocytosed Abs was examined under a laser scanning confocal microscope. As shown in Figure 3A, there were detectable Abs already in the cytoplasm of endplate chondrocytes and more Abs were co‐localized with COL‐II in the cytoplasm of endplate chondrocytes at 60 minutes post‐treatment. Furthermore, treatment with H_2_O_2_ significantly increased the contents of calcium and phosphate depositions, as assessed by Alizarin red S (Figure 3B and 3C) and von Kossa staining (Figure 3D,E), in endplate chondrocytes in a trend of dose‐dependence. In addition, treatment with either 2.0 mmol/L H_2_O_2_ or Abs for 7 days significantly increased the relative levels of ALP, OCN, RUNX2, and COL‐I mRNA transcripts in endplate chondrocytes and treatment with both further increased their mRNA transcripts in endplate chondrocytes (Figure 3F). However, treatment with the same dose of Abs did not cause cell death in our experimental system (Figure S1). Collectively, Abs from oxidative stressed endplate chondrocytes enhanced the H_2_O_2_‐mediated mineralization in endplate chondrocytes.

### 3.4 Abs from the oxidative stressed cells alter the levels of extracellular PPi in the supernatants of cultured endplate chondrocytes

**Figure 4:**
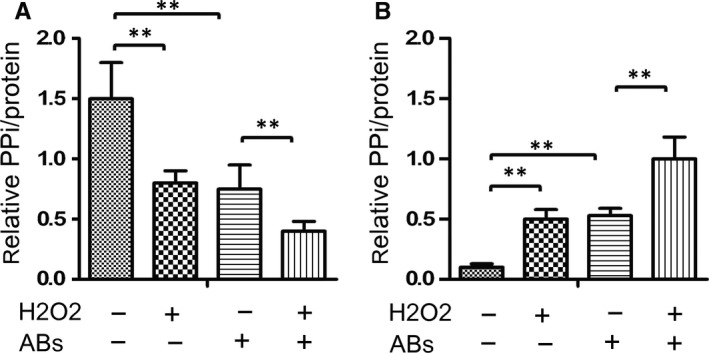
Oxidative stress and Abs promote the PPi metabolism in endplate chondrocytes. Endplate chondrocytes were treated with, or without, 2 mmol/L H_2_O_2_ and/or Abs for 24 hours. (A) and (B) The levels of PPi and Pi in the supernatants of cultured cells were measured. Data are expressed as the mean ± SD of each group of cells from three separate experiments. **P < 0.01 vs the control

It is wellknown that the levels of extracellular PPi and Pi are critical for mineralization in cartilage.^22^, ^23^ Accordingly, we tested the levels of extracellular PPi and Pi in the supernatants of cultured endplate chondrocytes following treatment with H_2_O_2_ and/or Abs. In comparison with the control cells, treatment with 2.0 mmol/L H_2_O_2_ or Abs significantly decreased the levels of PPi, but increased the levels of Pi in the supernatants of cultured endplate chondrocytes (Figure 4A,B). Treatment with both H_2_O_2_ and Abs further enhanced the changes in the supernatants of cultured endplate chondrocytes. These results support the notion that oxidative stress modulates calcification and promotes chondrocyte mineralization by increasing the contents of Pi.

### 3.5 Abs form the oxidative stressed cells alter the expression of PPi metabolism‐related genes in endplate chondrocytes

**Figure 5:**
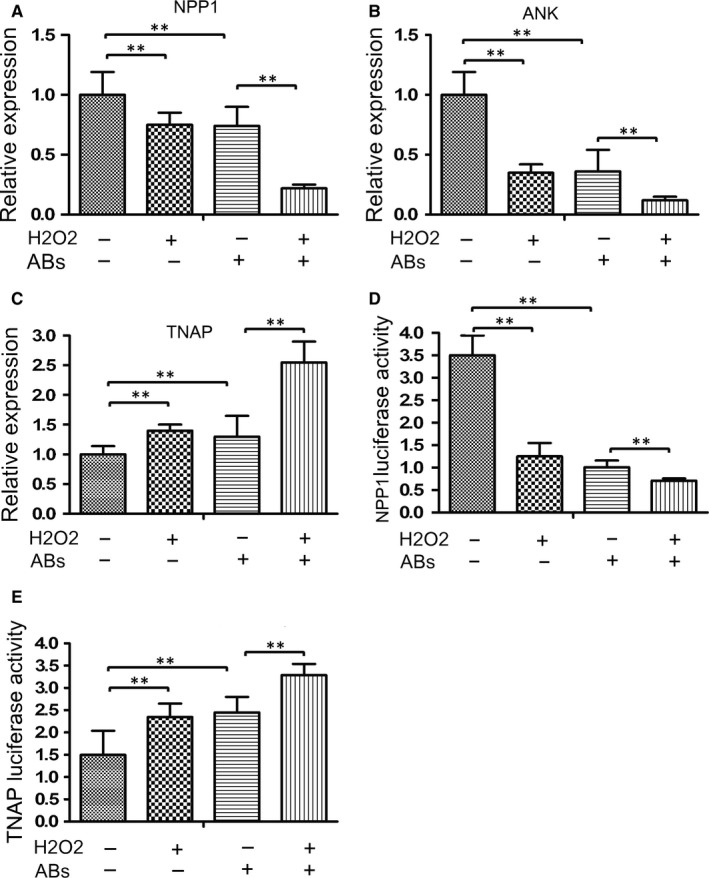
Oxidative stress and Abs modulate the expression of ENPP1, ANK and TNAP expression in endplate chondrocytes. Endplate chondrocytes were treated with, or without, H_2_O_2_ and/or Abs for 24 hours. (A‐C) The relative levels of ENPP1, ANK and TNAP mRNA transcripts in individual groups of cells were determined by quantitative RT‐PCR. Furthermore, endplate chondrocytes were transfected with plasmid for the ENPP1 or TNAP promoter‐controlled luciferase expression, together with plasmid for Renilla luciferase expression. Subsequently, the cells were treated with, or without, H_2_O_2_ and/or Abs for 24 hours. The relative levels of luciferase to Renilla luciferase activities were determined by Dual luciferase assays. (D‐E) The ENPP1 and TNAP promoter activities in individual groups of cells. Data are expressed as the mean ± SD of each group of cells from three separate experiments. **P < 0.01 vs the control

To understand the molecular mechanisms underlying the action of Abs in regulating the metabolism of PPi, we examined the relative levels of ENPP1, TNAP and ANK mRNA transcripts, which are primarily involved in PPi metabolism.^25^, ^26^ In comparison with that in the control cells, treatment with either 2.0 mmol/L H_2_O_2_ or Abs significantly decreased the relative levels of ENPP1 and ANK mRNA transcripts, but increased TNAP mRNA transcripts in endplate chondrocytes (Figure 5A‐C). Treatment with both H_2_O_2_ and Abs synergistically enhanced the changes in the relative levels of ENPP1, TNAP and ANK mRNA transcripts in endplate chondrocytes. Furthermore, luciferase assays revealed a similar pattern of the NPP1 and TNAP promoter activity in endplate chondrocytes following treatment with H_2_O_2_ and/or Abs, relative to that in the control cells (Figure 5D,E). Therefore, treatment with H_2_O_2_ or Abs significantly modulated the levels of PPi metabolism‐related gene expression in endplate chondrocytes.

## 4 DISCUSSION

In this study, our data indicated that oxidative stress significantly promoted mineralization in primarily cultured endplate chondrocytes from IVDs of rats, associated with increased levels of ALP, RUNX2, OCN and COL‐I expression in the endplate chondrocytes. Because oxidative stress can modulate the generation of extracellular vesicles, including Abs, we found that treatment with H_2_O_2_ significantly increased the production of Abs in endplate chondrocytes, probably contributing to mineral deposition. Actually, we found that Abs were effectively endocytosed and co‐localized with COL‐II in endplate chondrocytes in a short period after treatment with Abs. Furthermore, treatment with Abs significantly promoted mineral deposition and enhanced ALP, RUNX2, OCN and COL‐I expression in endplate chondrocytes in a dose‐dependent trend. More importantly, treatment with both H_2_O_2_ and Abs synergistically increased the relative levels of ALP, RUNX2, OCN and COL‐I expression in endplate chondrocytes. In addition, treatment with either H_2_O_2_ or Abs significantly decreased the levels of PPi while increased Pi in the supernatants of cultured endplate chondrocytes and treatment with both had strong regulatory effects, indicating that Abs enhanced the oxidative stress‐regulated PPi metabolism in endplate chondrocytes. Similarly, treatment with either H_2_O_2_ or Abs significantly decreased ENPP1 and ANK expression while increasing TNAP expression and treatment with both had synergistically regulatory effects in endplate chondrocytes. Moreover, similar patterns of ENPP1 and TNAP promoter activities were observed in the cells following treatment with H_2_O_2_ and/or Abs. Our novel data indicated that Abs contributed to the oxidative stress‐induced mineralization in endplate chondrocytes by regulating PPi metabolism. Conceivably, Abs from oxidative stressed chondrocytes may be new therapeutic targets for preventing matrix calcification and serve as a potential for treatment of CEP degeneration. We found that Abs were co‐localized with type II collagen in the cytoplasm of endplate chondrocytes in a short period after treatment with Abs, suggesting that Abs may be partially engulfed by endplate chondrocytes. However, we cannot exclude the possibility that Abs may mainly interact with molecules on the surface membrane of endplate chondrocytes. We are interested in further investigating the dynamic process of Abs in endplate chondrocytes.

It is well‐known that oxidative stress not only enhances matrix degradation and inflammation, but also reduces the number of active and functional cells in the IVD microenvironment.^18^, ^22^ Moreover, excessive ROS can modify the components of extracellular matrix proteins in the disc, damaging extracellular matrix in IVDs.^9^, ^27^ In this study, we found that treatment with H_2_O_2_ significantly increased mineralization in endplate chondrocytes in a dose‐ and time‐dependent manner. Our data support the notion that oxidative stress increases calcification.^28^

Endplate chondrocytes are embedded in a non‐vascular extracellular matrix in a hypoxic condition and generate energy mostly dependent on anaerobic glucose metabolism.^22^, ^29^, ^30^ Therefore, they are vulnerable to oxidative injury. It is well known that high levels of H_2_O_2_ induce apoptosis of many types of cells, including articular chondrocytes.^25^, ^28^, ^31^ Interestingly, apoptosis induced by ROS can promote the generation of Abs.^18^, ^32^, ^33^ In this study, we found that treatment with H_2_O_2_ significantly increased the generation of extracellular vesicles, particularly for Abs as the purified extracellular vesicles had a size of 1000‐3000 nm and contained high levels of Histone H3, but only little ARF6 and CD9. More importantly, we found that treatment with Abs significantly increased mineralization in a dose‐dependent trend and enhanced ALP, OCN, RUNX2 and COL‐I expression in endplate chondrocytes. Our findings extended previous observations that Abs form chondrocytes may contribute to the pathologic cartilage calcification in ageing individuals with osteoarthritis.^15^, ^17^ Interestingly, treatment with both H_2_O_2_ and Abs further increased mineralization in endplate chondrocytes. Therefore, such novel data demonstrated that Abs participated in the oxidative stress‐induced mineralization of endplate chondrocytes and the pathogenesis of IVD degeneration.

The balance between local mineralization promoters and inhibitors is crucial for the mineralization process in chondrocytes and is regulated by extracellular PPi and Pi contents.^15^, ^17^ While PPi potently inhibits the calcium/phosphate crystal nucleation and propagation.^35^ Pi is a major component of mineral hydroxyapatite (HA) and can promote mineralization.^36^ The process of PPi metabolism is mainly regulated by ENPP1, TNAP and ANK. ENPP1 inhibits HA crystals and serves as a physiological inhibitor of calcification by generating extracellular PPi.^37^, ^38^ TNAP, an enzyme that converts PPi to Pi, can decrease the extracellular PPi levels, and increase HA crystals.^39^, ^40^ ANK, a multiple‐pass transmembrane protein, can promote PPi secretion to inhibit mineralization.^38^ In this study, we found that treatment with either H_2_O_2_ or Abs significantly decreased the levels of PPi, but increased Pi in the supernatants of cultured endplate chondrocytes, accompanied by decreasing the levels of ENPP1 and ANK expression, but increasing TNAP expression as well as modulating the ENPP1 and TNAP promoter activities. More importantly, treatment with both further enhanced their regulatory effects in endplate chondrocytes. The increased TNAP expression and decreased ENPP1 and ANK expression by H_2_O_2_ and/or Abs co‐ordinately decreased PPi, but increased Pi to promote the process of calcification in endplate chondrocytes. It is well known that the EVs contain many components, including mRNAs, long non‐coding RNAs, rRNA, miRNAs or their fragments.^26^ We are interested in further investigating how oxidative stress regulates the expression of those regulators and PPi metabolism and which components in Abs can enhance the H2O2‐induced mineralization in chondrocytes.

**Figure 6:**
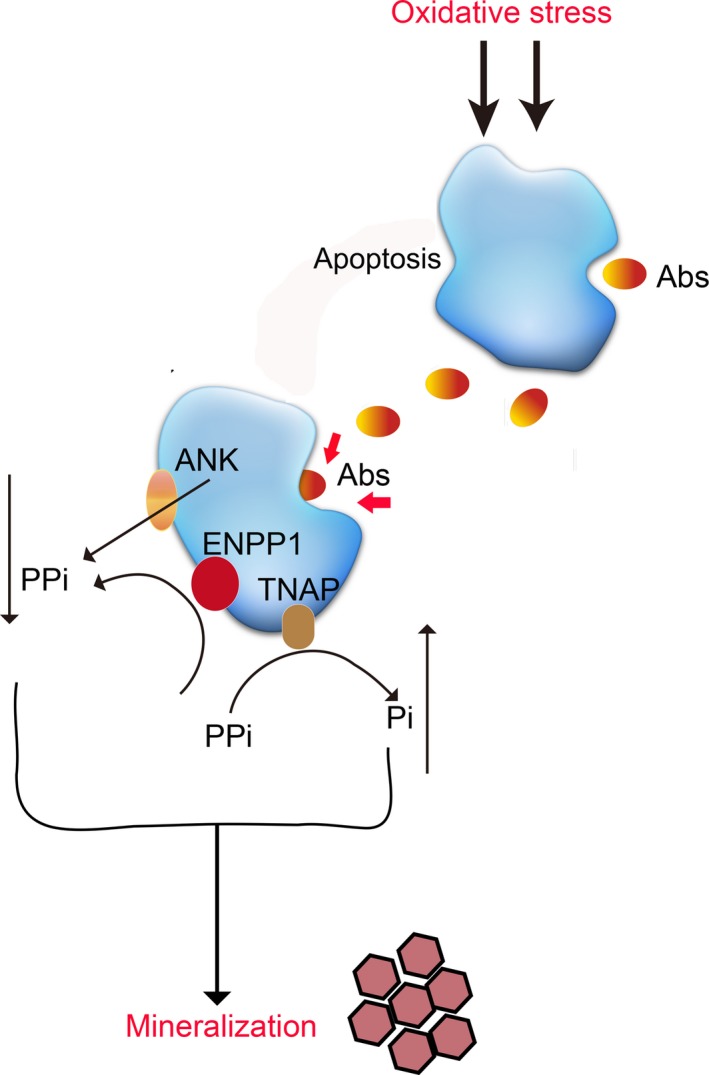
Schematic diagram illustrates the potential mechanisms by which oxidative stress induced the generation of Abs which might contribute to the mineralization by endplate chondrocytes. Abs increase PPi metabolism by modulating ENPP1, ANK and TNAP expression, contributing to the mineralization in endplate chondrocytes. Red arrows show that Abs may be partially engulfed by endplate chondrocytes

In conclusion, our data indicated that oxidative stress induced the generation of Abs that promoted the mineralization in primarily cultured endplate chondrocytes. Oxidative stress and its related Abs enhanced PPi metabolism by modulating ENPP1, ANK and TNAP expression, contributing to the mineralization in endplate chondrocytes (Figure 6). Our data suggest that both oxidative stress and its related Abs may be new therapeutic targets for intervention of CEP degeneration. Our findings may uncover new insights in the pathogenesis of CEP degeneration.

## CONFLICT OF INTEREST

The auths declare that there are no conflicts of interest associated with this study.

## AUTHOR'S CONTRIBUTION

Feng‐Lai Yua, Xia Li, and Li‐Jun Ren conceived the project and designed the experiments. Rui‐Sheng Xu and Jun‐Xing Ye collected and analysed the data. All auths developed analytical tools and wrote, edited and approved the final submission of the manuscript.
