# Supplementary material for: Apoptotic bodies from endplate chondrocytes enhance the oxidative stress‐induced mineralization by regulating PPi metabolism
Source: J Cell Mol Med. 2019 Mar 20;23(5):3665–75. doi: 10.1111/jcmm.14268 (PMC6484318; doi:10.1111/jcmm.14268)

Supplementary Figure 1. Abs do not alter the viability of endplate chondrocytes.

Endplate chondrocytes were treated with, or without, Abs (1 µg/ml) for 24 h and their viability was determined by CCk-8 assay using a specific kit, according to the manufacturer’s instruction. Data are expressed as the mean ± SD of each group of cells from three separate experiments.


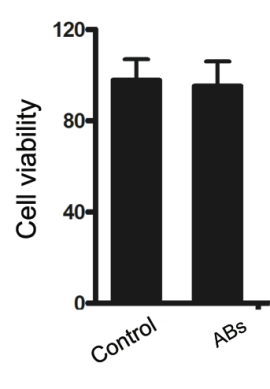

Supplement: Supplementary file 1 [file JCMM-23-3665-s001.docx]
